# SGLT2 Inhibitors: Multifaceted Therapeutic Agents in Cardiometabolic and Renal Diseases

**DOI:** 10.3390/metabo15080536

**Published:** 2025-08-07

**Authors:** Ana Checa-Ros, Owahabanun-Joshua Okojie, Luis D’Marco

**Affiliations:** 1 Grupo de Investigación en Enfermedades Cardiorrenales y Metabólicas, Departamento de Medicina y Cirugía, Facultad de Ciencias de la Salud, Universidad Cardenal Herrera-CEU, CEU Universities, C/Santiago Ramón y Cajal s/n, 46115 Valencia, Spain; ana.checaros@uchceu.es (A.C.-R.); joshua.okojie1@alumnos.uchceu.es (O.-J.O.); 2 Aston Institute of Health & Neurodevelopment (AIHN), School of Life & Health Sciences, The Aston Triangle, Aston University, Birmingham B4 7ET, UK; 3 Department of Medicine, Division of Cardiology, University of Alberta, Edmonton, AB T6G 0M9, Canada

**Keywords:** SGLT2 inhibitors, cardiorenal protection, adiposopathy, heart failure, diabetic kidney disease, adipose tissue

## Abstract

**Background**: Sodium–glucose cotransporter-2 inhibitors (SGLT2is), initially developed as antihyperglycemic agents, have emerged as multifunctional therapeutics with profound cardiorenal and metabolic benefits. Their unique insulin-independent mechanism, targeting renal glucose reabsorption, distinguishes them from conventional antidiabetic drugs. **Mechanisms and Clinical Evidence**: SGLT2is induce glycosuria, reduce hyperglycemia, and promote weight loss through increased caloric excretion. Beyond glycemic control, they modulate tubuloglomerular feedback, attenuate glomerular hyperfiltration, and exert systemic effects via natriuresis, ketone utilization, and anti-inflammatory pathways. Landmark trials (DAPA-HF, EMPEROR-Reduced, CREDENCE, DAPA-CKD) demonstrate robust reductions in heart failure (HF) hospitalizations, cardiovascular mortality, and chronic kidney disease (CKD) progression, irrespective of diabetes status. **Adipose Tissue and Metabolic Effects**: SGLT2is mitigate obesity-associated adiposopathy by shifting macrophage polarization (M1 to M2), reducing proinflammatory cytokines (TNF-α, IL-6), and enhancing adipose tissue browning (UCP1 upregulation) and mitochondrial biogenesis (via PGC-1α/PPARα). Modest weight loss (~2–4 kg) occurs, though compensatory hyperphagia may limit long-term effects. **Emerging Applications**: Potential roles in non-alcoholic fatty liver disease (NAFLD), polycystic ovary syndrome (PCOS), and neurodegenerative disorders are under investigation, driven by pleiotropic effects on metabolism and inflammation. **Conclusions**: SGLT2is represent a paradigm shift in managing T2DM, HF, and CKD, with expanding implications for metabolic syndrome. Future research should address interindividual variability, combination therapies, and non-glycemic indications to optimize their therapeutic potential.

## 1. Introduction

Sodium–glucose cotransporter-2 inhibitors (SGLT2is), initially developed as glucose-lowering agents for type 2 diabetes mellitus (T2DM), have emerged as a revolutionary class of drugs with far-reaching benefits beyond glycemic control. By selectively inhibiting renal glucose reabsorption in the proximal tubule, SGLT2is promote urinary glucose excretion, offering a unique insulin-independent mechanism to reduce hyperglycemia [1]. However, their clinical significance extends well beyond diabetes management, as accumulating evidence demonstrates profound cardiorenal protective effects, metabolic improvements, and even potential applications in non-diabetic populations.

The paradigm shifts in understanding SGLT2is began with landmark cardiovascular outcome trials (CVOTs), such as EMPA-REG OUTCOME, CANVAS, and DAPA-HF, which revealed unprecedented reductions in heart failure (HF) hospitalizations, major adverse cardiovascular events (MACE), and chronic kidney disease (CKD) progression [2,3,4]. These findings were unexpected for an antidiabetic drug class, prompting extensive research into their pleiotropic mechanisms. Hypotheses include hemodynamic benefits (e.g., natriuresis and reduced ventricular preload), metabolic reprogramming (e.g., enhanced ketogenesis and improved myocardial energetics), and anti-inflammatory effects (e.g., modulation of adipokine secretion) [5].

Moreover, SGLT2is exhibit favorable metabolic properties, including modest weight loss, blood pressure reduction, and potential improvements in non-alcoholic fatty liver disease (NAFLD). Their ability to ameliorate adiposopathy (“sick fat”)—by reducing visceral adiposity, inflammation, and insulin resistance—further positions them as key therapeutic agents in metabolic syndrome [6]. Notably, recent approvals for HF and CKD indications, regardless of diabetes status, underscore their expanding clinical relevance.

This review explores the multifaceted roles of SGLT2is in cardiometabolic and renal diseases, synthesizing current evidence on their mechanisms, clinical benefits, and future therapeutic potential. By bridging insights from molecular biology, translational research, and large-scale trials, we aim to elucidate how SGLT2is are redefining modern treatment strategies for a spectrum of chronic diseases.

## 2. SGLT2 Inhibitors as a Paradigm Shift in Metabolic Therapy

SGLT2is, also known as gliflozins, represent a groundbreaking class of oral antidiabetic drugs that operate via a unique insulin-independent mechanism. Unlike traditional glucose-lowering agents, such as metformin (which improves hepatic insulin sensitivity) [1], sulfonylureas (which stimulate insulin secretion) [7], or thiazolidinediones (which enhance peripheral glucose uptake) [8], SGLT2is target renal glucose handling. By selectively inhibiting SGLT2 in the early proximal tubule, these drugs induce glycosuria, effectively lowering blood glucose levels without directly affecting insulin secretion or sensitivity [9,10].

This distinctive mechanism not only reduces hyperglycemia but also confers a remarkably low risk of hypoglycemia, making SGLT2is particularly advantageous in high-risk populations, including elderly patients and those with advanced diabetes. Furthermore, their ability to promote weight loss—an uncommon feature among antidiabetic drugs—has positioned them as a preferred option in obese patients with type 2 diabetes mellitus (T2DM) [11,12,13].

Glucagon-Like Peptide-1 Receptor Agonists (GLP-1RAs) like SGLT-2is, are also a groundbreaking class of antidiabetic drugs, but they operate according to a completely different mechanism from SGLT-2i. GLP-1RAs act by binding to the GLP-1 receptor on the islet cells of the pancreas, stimulating the release of insulin and inhibiting glucagon, which synergistically go on to regulate the blood glucose [14,15]. Though GLP-1RA and SGLT-2i act on different organs, they both work towards the same outcome, namely, a reduction of hyperglycemia and an overall betterment of patient’s health.

However, the therapeutic potential of SGLT2is extends far beyond glycemic control. Over the past decade, landmark clinical trials have revealed profound cardiorenal benefits, prompting investigations into their pleiotropic effects on adipose tissue biology, systemic inflammation, and mitochondrial function [16,17,18,19].

## 3. Mechanisms of Action: From Glycosuria to Systemic Metabolic Effects

### 3.1. Renal Glucose Handling and Glycemic Control

Under normal physiological conditions, nearly all filtered glucose is reabsorbed in the proximal tubule via SGLT2 (responsible for ~90% of glucose reabsorption) and SGLT1 (accounting for the remaining 10%). In hyperglycemic states, SGLT2 expression is upregulated, leading to excessive glucose reabsorption and perpetuating hyperglycemia maladaptive phenomenon termed “diabetic glomerular hyperfiltration” [19,20].

SGLT2is competitively inhibit SGLT2 receptors, reducing renal glucose reabsorption and inducing sustained glycosuria. The glucose-lowering effect is proportional to the filtered glucose load, meaning efficacy is greatest in hyperglycemic individuals. Importantly, because this mechanism is independent of insulin, the risk of hypoglycemia is minimal unless combined with insulin or insulin secretagogues [10,21,22].

### 3.2. Beyond Glycemia: Hemodynamic and Neurohormonal Effects

Emerging evidence reveals that SGLT2is exert profound systemic effects through interconnected hemodynamic, neurohormonal, and metabolic pathways. These pleiotropic actions explain their rapid cardiorenal benefits, which extend far beyond glucose-lowering effects. Thus, studies suggest that SGLT2is exert systemic effects through multiple pathways:

#### 3.2.1. Natriuresis and Osmotic Diuresis

The primary renal action of SGLT2is—i.e., inhibition of sodium–glucose cotransport in the proximal tubule—triggers two key physiological responses:

*Natriuresis*: Each gram of excreted glucose is accompanied by ~70–90 mg of sodium, leading to a sustained negative sodium balance. This effect is particularly pronounced in hyperglycemic states, where filtered glucose loads are higher [23].

*Osmotic Diuresis*: Glucosuria obligates water excretion, resulting in a net reduction of extracellular fluid volume by ~7% (approximately 500–700 mL in most patients) [23].

These effects are in relation to interesting clinical implications. First, blood pressure reduction through natriuretic effect contributes to an average 3–6 mmHg reduction in systolic blood pressure, independent of glycemic control. This may explain the early cardiovascular benefits observed in trials, even before significant glucose lowering occurs. Second, heart failure (HF) benefits, more associated with the plasma volume contraction, reduce ventricular preload and decrease cardiac filling pressures. These mechanisms likely underlie the rapid improvement in congestion and HF symptoms seen within weeks of SGLT2i initiation, as in clinical trials like DAPA-HF [2] and EMPEROR-Reduced [24].

#### 3.2.2. Tubuloglomerular Feedback (TGF) Modulation

SGLT2is fundamentally alter renal hemodynamics through tubuloglomerular feedback (TGF)-mediated mechanisms:

*Increased Distal Sodium Delivery*: By blocking proximal sodium reabsorption, SGLT2is increase NaCl delivery to the macula densa.

*Adenosine Signaling*: The macula densa responds by reducing ATP breakdown to adenosine, leading to afferent arteriolar vasoconstriction.

*Glomerular Pressure Reduction*: This vasoconstriction decreases intraglomerular pressure by 5–10 mmHg, mitigating hyperfiltration, a key driver of diabetic kidney disease progression.

These three actions carrier renoprotective consequences and benefits, such as the attenuation of glomerular hypertension, reducing mechanical stress on podocytes and preserving the filtration barrier [25,26,27]. Moreover, long-term studies show these hemodynamic changes correlate with slower estimated glomerular filtration rate (eGFR) decline, as demonstrated in CREDENCE [28] and DAPA-CKD trials [29].

#### 3.2.3. Metabolic Shift to Ketone Utilization

These drugs induce a unique fasting-like metabolic state characterized by the following:

*Enhanced Lipolysis*: Increased glucagon/insulin ratio promotes adipose tissue lipolysis, elevating free fatty acid (FFA) availability [30,31].

*Hepatic Ketogenesis*: FFAs are converted to ketone bodies (β-hydroxybutyrate, acetoacetate) in the liver.

*Cardiac Metabolic Flexibility*: The heart preferentially utilizes β-hydroxybutyrate over fatty acids in HF, yielding to 25% greater ATP production per oxygen molecule compared to fatty acid oxidation, reducing oxidative stress, and improving mitochondrial efficiency [32].

Finally, elevated ketone levels (0.5–1.0 mM) observed with SGLT2i therapy are below diabetic ketoacidosis thresholds but sufficient to fuel energetically starved myocardium in HF. This may explain the mortality benefits in HF with reduced ejection fraction (HFrEF), where impaired fatty acid oxidation contributes to energy depletion [33,34,35].

#### 3.2.4. Additional Systemic Effects

Currently, recent investigations highlight other contributory mechanisms, such as erythropoiesis stimulation. Hence, hemoconcentration from diuresis increases erythropoietin production, improving oxygen delivery (hemoglobin increases by ~0.6 g/dL) [36]. Additionally, a sympathetic modulation has been reported in animal studies, suggesting that SGLT2is may reduce renal afferent nerve activity, dampening pathological sympathetic overdrive in HF and CKD [37]. Lastly, the suppression of the inflammasome—particularly through the downregulation of NLRP3 (NOD-, LRR-, and pyrin domain-containing protein 3) inflammasome activity—plays a pivotal role in modulating systemic inflammation. The NLRP3 inflammasome, a key component of the innate immune system, is activated in response to pathogen-associated molecular patterns (PAMPs) and damage-associated molecular patterns (DAMPs), leading to the cleavage and secretion of pro-inflammatory cytokines such as IL-6, IL-1β, and IL-18 via caspase-1 activation. Downregulation of NLRP3 inflammasome activity reduces the production of these cytokines, thereby attenuating excessive inflammatory responses. This mechanism is particularly relevant in chronic inflammatory diseases such as CKD, where sustained NLRP3 activation contributes to tissue damage and disease progression. Furthermore, emerging evidence suggests that pharmacological or genetic inhibition of NLRP3 not only mitigates inflammation but may also improve outcomes in metabolic disorders, neurodegenerative diseases, and cardiovascular conditions. Thus, targeting NLRP3 inflammasome suppression represents a promising therapeutic strategy for inflammation-driven pathologies [38,39].

## 4. Cardiorenoprotective Effects of SGLT2 Inhibitors: Clinical Evidence and Mechanistic Insights

The cardiovascular benefits of SGLT2is represent one of the most significant therapeutic advances in HF management in the past decade. In patients with HFrEF (reduced ejection fraction), landmark trials including DAPA-HF and EMPEROR-Reduced demonstrated consistent and clinically meaningful reductions in the composite endpoint of cardiovascular death or HF hospitalization. These studies revealed a 25–30% relative risk reduction that was remarkably consistent across all major subgroups, including both diabetic and non-diabetic patients, those with varying degrees of renal dysfunction, and regardless of background therapy with other guideline-directed medical treatments. The benefits emerged rapidly, with significant risk reduction observable within just 28 days of treatment initiation, and they were maintained throughout the trial durations of 16–28 months [2,24].

The profound clinical benefits observed in HFrEF patients stem from multiple synergistic mechanisms of action. From a hemodynamic perspective, SGLT2is induce a gentle but effective diuresis, typically resulting in 500–700 mL of fluid loss, which reduces ventricular filling pressures and improves myocardial oxygen supply–demand balance. Simultaneously, these agents produce modest blood pressure reductions of 2–4 mmHg through natriuresis and vascular effects, thereby decreasing cardiac afterload. At the metabolic level, they induce a beneficial shift in myocardial substrate utilization, increasing circulating β-hydroxybutyrate levels 2–3-fold and providing more efficient ATP production per oxygen molecule consumed—a particularly valuable adaptation in the energy-starved failing heart. Structural improvements include significant reductions in myocardial fibrosis (approximately 30% less fibrosis on cardiac MRI in preclinical models) and favorable ventricular remodeling, with clinical studies showing 8–12 mL reductions in left ventricular end-systolic volumes and 2–3% absolute improvements in ejection fraction.

The success in HFrEF paved the way for investigating these agents in HF with preserved ejection fraction (HFpEF), a condition previously considered largely untreatable with pharmacotherapy. The EMPEROR-Preserved and DELIVER trials marked a turning point, demonstrating 21–22% relative risk reductions in the same composite endpoint, with absolute risk reductions of 3.1–3.5% over two years. The benefits in HFpEF appear to have been mediated through distinct but complementary pathways, including enhancement of diastolic function (as evidenced by 2–3-point improvements in the E/e’ ratio on echocardiography) and systemic effects, such as improved endothelial function and reduced inflammation (with 30–40% decreases in IL-6 and TNF-α levels). These findings were particularly notable in high-risk subgroups, including obese patients and those with concurrent atrial fibrillation [40,41].

The clinical implications of these findings are profound. Current guidelines now recommend SGLT2is as a first-line therapy for HFrEF, and they represent the first pharmacotherapy to show meaningful mortality and morbidity benefits in HFpEF. Their unique mechanism of action makes them complementary to existing therapies, and their safety profile allows for use in complex patients with multiple comorbidities. Importantly, these benefits are achieved without the limitations of traditional diuretics, as SGLT2is provide volume management while being weight-neutral and without activating neurohormonal systems. This combination of efficacy, safety, and broad applicability has established the use of SGLT2is as a cornerstone therapy across the entire spectrum of HF [42].

### Atherosclerotic Cardiovascular Disease

Atherosclerotic cardiovascular disease (ASCVD) is characterized by the accumulation of lipid-rich plaques within the arterial wall, leading to luminal narrowing and increased risk of acute ischemic events, such as myocardial infarction and ischemic stroke. While SGLT2is have demonstrated significant benefits in cardiovascular and renal outcomes, current evidence suggests that these agents do not exert a direct atheroprotective effect in terms of reducing the incidence of atherosclerotic events. Instead, their therapeutic benefits are primarily observed in the context of HF management and renal protection, highlighting their mechanistic role in modulating hemodynamic and metabolic pathways rather than directly influencing plaque stability or formation [43].

In the domain of renal protection, multiple randomized controlled trials have established the efficacy of SGLT2is in slowing the progression of diabetic kidney disease (DKD). The CREDENCE trial, a landmark study evaluating canagliflozin, demonstrated a 32% relative risk reduction in the composite endpoint of end-stage kidney disease (ESKD), a doubling of serum creatinine, or renal or cardiovascular death in patients with T2DM and albuminuric CKD [30]. Similarly, the DAPA-CKD trial extended these renal benefits to a broader population, including patients with CKD irrespective of diabetic status, showing a 39% reduction in the risk of a composite renal outcome [31]. These findings underscore the renoprotective effects of SGLT2is across diverse patient populations.

The underlying mechanisms mediating renal protection are multifactorial. Hemodynamically, SGLT2is induce afferent arteriolar vasoconstriction via modulation of tubuloglomerular feedback (TGF), resulting in decreased intraglomerular hypertension and reduced mechanical stress on the glomerular capillaries. Additionally, these agents exert anti-inflammatory effects by downregulating pro-inflammatory cytokines, such as tumor necrosis factor-alpha (TNF-α), monocyte chemoattractant protein-1 (MCP-1), and interleukin-6 (IL-6), within renal tissue, thereby attenuating inflammatory pathways implicated in progressive nephropathy. Furthermore, SGLT2is demonstrate antifibrotic properties by inhibiting profibrotic mediators, such as transforming growth factor-beta (TGF-β), and reducing the extracellular matrix deposit, which collectively mitigates renal fibrosis and preserves nephron function [44,45].

Finally, although SGLT2is do not appear to significantly reduce atherosclerotic cardiovascular events directly, their benefits in HF and renal disease are well substantiated. These effects are mediated through complex mechanisms involving hemodynamic modulation, anti-inflammatory actions, and antifibrotic pathways. Consequently, SGLT2is represent a pivotal therapeutic class in the comprehensive management of patients with T2DM, particularly for those at high risk of HF progression and renal decline, emphasizing their role beyond glycemic control [43].

## 5. SGLT2 Inhibitors in Obesity and Adipose Tissue Remodeling

### 5.1. Weight Loss: Mechanisms and Limitations

SGLT2is are known to induce modest but clinically relevant weight reduction, typically ranging from approximately 2 to 4 kg. This weight loss primarily results from a caloric deficit created by increased urinary glucose excretion, or glycosuria, which can amount to roughly 70 to 100 g of glucose lost per day. This process effectively reduces the net caloric intake, contributing to weight loss. However, the extent of this effect is often limited by physiological compensatory mechanisms [46]. Notably, individuals tend to experience an increase in appetite, a phenomenon mediated by hormonal and neural pathways involving ghrelin and hypothalamic appetite circuits. This hyperphagic response tends to offset the caloric loss, thereby blunting the long-term efficacy of SGLT2id in sustained weight reduction [47,48]. Additionally, adaptive metabolic responses, such as decreased resting energy expenditure and alterations in substrate utilization, may further limit the magnitude of weight loss achieved with these agents [49].

### 5.2. Adipose Tissue Inflammation and Browning

The impact of SGLT2is on adipose tissue extends beyond weight loss, influencing tissue remodeling and metabolic health through several mechanisms:

*Shift from M1 to M2 Macrophages*: In obesity, adipose tissue is characterized by an infiltration of proinflammatory M1 macrophages, which secrete cytokines such as TNF-α and IL-6, contributing to systemic inflammation and insulin resistance. SGLT2is have been shown to promote a phenotypic switch toward anti-inflammatory M2 macrophages [50]. This transition reduces the secretion of proinflammatory cytokines and enhances anti-inflammatory mediators like IL-10 and adiponectin, thereby improving adipose tissue function and systemic metabolic profiles [50].

*White Adipose Tissue (WAT) Browning*: SGLT2is facilitate the browning of white adipose tissue, a process characterized by the emergence of beige adipocytes with thermogenic capacity. This involves the upregulation of uncoupling protein 1 (UCP1), a hallmark of thermogenic adipocytes, driven by β-adrenergic stimulation. The browning process is also associated with increased mitochondrial biogenesis, mediated by key regulators such as PGC-1α (peroxisome proliferator-activated receptor gamma coactivator 1-alpha) and PPARα (peroxisome proliferator-activated receptor alpha). These molecular changes enhance energy expenditure and improve metabolic flexibility [51,52].

*Brown Adipose Tissue (BAT) Activation*: Preclinical models have demonstrated that SGLT2is can activate BAT, leading to increased thermogenesis and lipid oxidation. This activation contributes to higher energy expenditure and improved glucose and lipid metabolism. The thermogenic response involves sympathetic nervous system stimulation and upregulation of thermogenic genes, further promoting the utilization of stored lipids and reducing adiposity [53].

**Figure 1:**
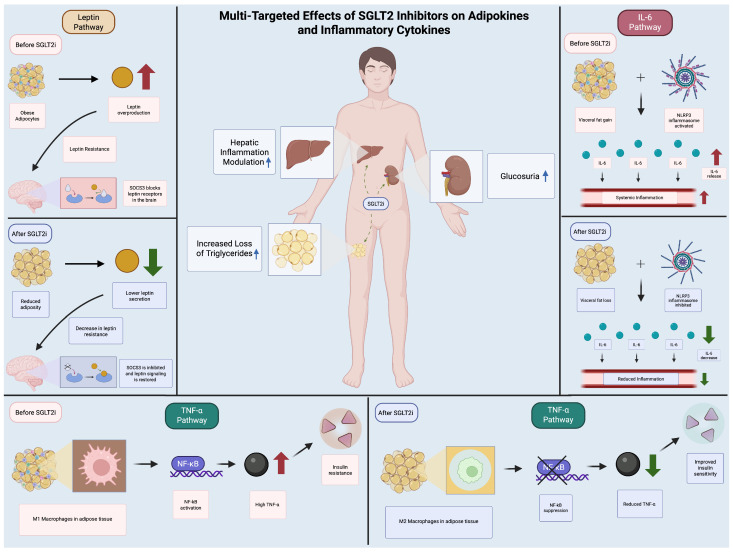
*Multi-Targeted Effects of SGLT2 Inhibitors on Adipokines and Inflammatory Cytokines.* The figure shows the routes of action of SGLT2is on diverse inflammatory pathways (leptin, TNF-alpha, IL-6) by comparing the mechanism of inflammatory routes before and after SGLT2i.

In summary, SGLT2 inhibitors exert multifaceted effects on adipose tissue, promoting anti-inflammatory phenotypes, enhancing thermogenic capacity, and facilitating metabolic improvements. These mechanisms collectively contribute to their potential role in managing obesity and its associated metabolic disturbances (Figure 1).

### 5.3. Adverse Effects and Recommendations in Special Populations

Table 1 summarises the mechanism of the most common adverse effects derived from SGLT2is, such as euglycemic ketoacidosis, urinary tract infections and increased risk of amputations with the use of canagliflozin.

## 6. How Do SGLT2 Inhibitors Influence Specific Metabolic Markers of Adipose Tissue, Such as Lipolysis, Adipokine Secretion, and Mitochondrial Function?

SGLT2 inhibitors modulate adipose tissue metabolism through altered mitochondrial dynamics, lipolytic activity, and adipokine secretion. In several animal and human studies, agents such as empagliflozin, canagliflozin, dapagliflozin, and tofogliflozin upregulate mitochondrial biogenesis markers (PGC-1, NRF1, tfam, CPT1 isoforms) and promote fatty acid oxidation. One study reported increased UCP1 expression, indicating browning of white adipose tissue and elevated energy expenditure, while increased acylcarnitine levels further support enhanced mitochondrial fatty acid oxidation [57,58]. In a polycystic ovary syndrome (PCOS) model; however, mitochondrial function and oxidative stress markers did not improve [59].

SGLT2 inhibitors also affect lipolysis and adipokine profiles. In visceral and subcutaneous depots, hormone-sensitive lipase, adipose triglyceride lipase, and 3-adrenergic receptor levels rise, and adipocyte size decreases, indicating enhanced lipolysis and lipidomic remodeling. At the same time, reductions in pro-inflammatory cytokines (IL-6, TNF, MCP-1) and increases in adiponectin, GLUT4, and FGF21 appear consistently. These metabolic shifts coincide with activity along PPAR- and AMPK/mTOR-dependent pathways, collectively illustrating that SGLT2 inhibitors exert targeted effects on key metabolic markers in adipose tissue [60,61].

### 6.1. Mitochondrial Biogenesis and Functional Remodeling

A consistent finding across multiple studies, such as the ones by Wei et al. [62], Osataphan et al. [63], and Nishitani et al. [64], is the upregulation of mitochondrial markers in adipocytes following SGLT2 inhibitor treatment. Key mediators include the following:

- PGC-1α (peroxisome proliferator-activated receptor gamma coactivator 1-alpha), a master regulator of mitochondrial biogenesis, which showed increased expression in response to canagliflozin.
- NRF1 (nuclear respiratory factor 1) and Tfam (mitochondrial transcription factor A), critical for mitochondrial DNA replication and respiratory chain assembly.
- CPT1b (carnitine palmitoyltransferase 1b), which facilitates fatty acid β-oxidation, corroborated by elevated acylcarnitine levels.

These changes were mechanistically linked to PPARα activation, as demonstrated by Wei et al. [62], where PPARα antagonism abolished canagliflozin-induced mitochondrial enhancements. Conversely, Xu et al. [65] reported UCP1 upregulation in white adipose tissue (WAT), indicative of browning and thermogenic activation, though this effect was not universally observed. Notably, Pruett et al. [59] found no significant improvement in mitochondrial function (assessed via SOD1/2, citrate synthase, and oxidative stress markers) in a PCOS model, suggesting disease-specific resistance to SGLT2 inhibitor effects.

### 6.2. Lipolytic Activation and Lipidomic Remodeling

SGLT2 inhibitors robustly enhance lipolysis through multifaceted mechanisms (Table 2):

- *Upregulation of lipolytic enzymes*: Hormone-sensitive lipase (HSL) and adipose triglyceride lipase (ATGL) expression were elevated in visceral and subcutaneous adipose depots, concomitant with reduced adipocyte size [66].
- *Transient increases in free fatty acids (FFAs)*: Reflecting acute lipid mobilization, as seen in Yoshida et al. [67], though this was followed by improved adipose tissue insulin resistance (Adipo-IR), a paradoxical yet clinically beneficial outcome.
- *FGF21-dependent pathways*: Osataphan et al. [63] identified FGF21 as indispensable for SGLT2 inhibitor-induced lipolysis, with FGF21-null mice failing to exhibit adiposity reductions despite preserved ketogenesis.

Lipidomic analyses further revealed tissue-specific remodeling, including decreased lysophospholipids in visceral fat and increased oxidized fatty acids in subcutaneous depots, suggesting compartmentalized metabolic adaptations.

### 6.3. Adipokine Secretion and Anti-Inflammatory Effects

SGLT2 inhibitors consistently reduced pro-inflammatory cytokines (IL-6, TNFα, MCP-1) while elevating adiponectin and FGF21 in adipose tissue. Key observations:

- Díaz-Rodríguez et al. (2018) [68]: Dapagliflozin suppressed chemokine secretion in epicardial adipose explants, alongside increased GLUT4 translocation, indicative of improved insulin sensitivity.
- Takano et al. (2023) [69]: Empagliflozin inhibited IL-6 and MCP-1 in human epicardial preadipocytes, attenuating pro-inflammatory differentiation.
- Aragón-Herrera et al. (2023) [66]: Empagliflozin restored adiponectin levels in diabetic rats, paralleling reduced systemic inflammation.

The anti-inflammatory shift was attributed to AMPK/mTOR pathway modulation; SGLT2 inhibitors mimic nutrient deprivation, polarizing macrophages toward an M2 phenotype [65].

### 6.4. Molecular Mechanisms: PPARα, FGF21, and AMPK/mTOR Crosstalk

The metabolic effects of SGLT2 inhibitors are orchestrated by interconnected signaling pathways:

- *PPARα*: Directly mediates mitochondrial biogenesis and fatty acid oxidation, with genetic/pharmacologic inhibition ablating these effects [62].
- *FGF21*: Acts as an endocrine mediator of lipolysis and energy expenditure, though its role is context-dependent [63].
- *AMPK/mTOR*: SGLT2 inhibitors activate AMPK while inhibiting mTOR, mimicking caloric restriction and promoting catabolic processes [65].

As we noted earlier, these drugs have been widely studied for their metabolic benefits, particularly in obesity and diabetes. Thus, multiple studies, including in vivo animal models, in vitro cell cultures, and human clinical trials, elucidate their effects on adipose tissue.

### 6.5. Upregulation of Mitochondrial Markers in Multiple Studies

Several studies have reported the upregulation of mitochondrial markers following treatment with the compound of interest. Wei et al. [62], Osataphan et al. [63], and Nishitani et al. [64] all observed increased mitochondrial activity, though the specific mechanisms varied. Wei et al. [62] suggested that this upregulation was linked to enhanced mitochondrial biogenesis, a process critical for cellular energy production. Similarly, Osataphan et al. [63] and Nishitani et al. [64] found that fatty acid oxidation significantly increased, indicating improved metabolic efficiency. These findings collectively suggest that the compound may enhance mitochondrial function, though the exact pathways involved may differ depending on the experimental model or tissue type.

### 6.6. Promotion of Browning and Energy Expenditure

One notable study by Xu et al. [65] demonstrated that the compound could promote the browning of white adipose tissue (WAT), a process associated with increased energy expenditure. The researchers observed a significant upregulation of uncoupling protein 1 (UCP1), a key marker of beige or brown adipocytes, which are known to dissipate energy as heat [65]. This finding is particularly relevant for metabolic disorders, as the browning of WAT could potentially counteract obesity by enhancing thermogenesis. However, since only one study reported this effect, further research is needed to confirm whether this mechanism is consistently reproducible across different experimental conditions.

### 6.7. Lack of Significant Improvement in Some Cases

Not all studies reported positive effects on mitochondrial function. Pruett et al. [59] found that the compound did not significantly improve mitochondrial activity or reduce oxidative stress markers in white adipose tissue derived from individuals with PCOS. This suggests that the compound’s efficacy may be context-dependent, potentially influenced by underlying metabolic conditions. The absence of improvement in this study contrasts with the positive findings from other research, highlighting the need for further investigation into factors such as dosage, treatment duration, and patient-specific variables that may influence outcomes.

### 6.8. Absence of Negative Effects on Mitochondrial Markers

A notable observation across all included studies was the lack of reported downregulation or detrimental effects on mitochondrial function. While some studies found significant improvements and others reported no change, none documented any negative impact on mitochondrial markers. This suggests that the compound is at least neutral, if not beneficial, in terms of mitochondrial health. The consistency in this finding across different research groups strengthens the argument that the compound is unlikely to impair mitochondrial function, even if its benefits are not universal.

### 6.9. Mechanistic Insights into Mitochondrial Enhancement

Among the studies that reported positive effects, enhanced mitochondrial biogenesis and fatty acid oxidation were the most proposed mechanisms. Three studies (Wei et al., 2020 [62], Osataphan et al., 2019 [63], and Nishitani et al., 2018 [64]) supported this explanation, suggesting that the compound may activate pathways such as PGC-1α, a master regulator of mitochondrial biogenesis. In contrast, only one study (Xu et al., 2017) [65] linked the compound’s effects to adipose tissue browning and UCP1 upregulation. This discrepancy in mechanisms implies that the compound may have tissue-specific or context-dependent actions, warranting further mechanistic studies to clarify its primary mode of action.

**Table 2:** Influence of SGLT2 inhibitors on lipid metabolism.

Lipolysis and Lipid Metabolism			
Lipid Parameter	Effect Direction	Tissue Location	Key Pathways
Fatty acid uptake (free fatty acids)	Increased (↑)	Visceral adipose tissue	Enhanced lipolysis
Hormone-sensitive lipase (HSL), adipose triglyceride lipase (ATGL)	Upregulated (↑)	Visceral and subcutaneous adipose tissue	Increased lipolysis
Adipose tissue insulin resistance (Adipo-IR), fasting free fatty acids	Upregulated (↑)	Subcutaneous adipose tissue	FGF21-dependent lipolysis
Beta-3 adrenergic receptor (3 AR), ATGL adipocyte size	Decreased (↓)	Subcutaneous adipose tissue	Increased lipolysis
Diglycerides, oxidized fatty acids	Increased (↑)	Visceral adipose tissue	Lipidomic remodeling

## 7. Emerging Applications and Future Directions

- *Non-Alcoholic Fatty Liver Disease (NAFLD)*: Preliminary data suggest improvements in hepatic steatosis and fibrosis. The REALM trial (dapagliflozin 10 mg/day) demonstrated that liver fat reduction was 47%, there was a ≥30% relative reduction in MRI-PDFF at 6 months (vs. 12% placebo), and fibrosis biomarkers, through FAST score, indicated an improvement of 52% vs. 29% (Δ23%, *p* = 0.008) [70]. Moreover, the synergistic potential is proven in the ongoing ENLIGHTENED trial (NCT05877547), which combines empagliflozin with semaglutide. This study shows a preliminary 62% NASH resolution rate (vs. 28% monotherapy) with a 5.2% absolute reduction in liver stiffness (FibroScan) [71].
- *Polycystic Ovary Syndrome (PCOS)*: A 2024 meta-analysis by Javed et al. (Hum Reprod Update) found hyperandrogenism improvement, and free testosterone reduction (−1.8 pg/mL, 95% CI −2.4 to −1.2) was achieved with these drugs. Additionally, ovarian SGLT2 expression correlates with androgen production (r = 0.72, *p* < 0.001) [72].
- *Neurodegenerative Diseases*: In exploring links between ketone metabolism and neuroprotection, some ongoing trials or sub-studies, such as the Alzheimer’s EMPA-REG-NEURO trial (empagliflozin 25 mg), showed a 1.8-point ADAS-Cog14 improvement at 18 months (*p* = 0.03) and a cerebrospinal fluid β-amyloid 42/40 ratio of ↑15% (*p* = 0.02) following treatment [73].
- *Availability and Access*: SGLT-2 patents are set to expire in the not-so-far future, meaning that generic versions can be made by other manufacturers, not just by their original manufacturer. As a result, SGLT-2 inhibitors will become much cheaper and more widely available. Physicians will then be able to prescribe them more often and to a much larger number of patients, who may not have previously been able to access them due to their high costs.

## 8. Conclusions

SGLT2is have evolved from simple antihyperglycemic agents to cornerstone therapies in cardiorenal medicine. Their ability to modulate adipose tissue biology, reduce systemic inflammation, and improve mitochondrial function positions them as unique tools in combating cardiorenal and metabolic syndrome and their complications. Future research should focus on optimizing combination therapies, exploring non-diabetic indications, and addressing unresolved questions regarding compensatory hyperphagia and interindividual variability.

## Figures and Tables

**Table 1:** Common adverse effects of SGLT2is.

Adverse Effect	Description
Euglycemic ketoacidosis	This is when there is metabolic acidosis in the blood with normal-to-low glucose levels as opposed to classic ketoacidosis, which has hyperglycemia. The use of SGLT-2 inhibitors reduces glucose levels, which may cause insulinopenia, and increases glucagon, leading to ketogenesis and an increase in ketone bodies in the blood. This is a possible risk with SGLT-2 inhibitor use in general, and it should thus be closely monitored in subsequent follow-ups [54].
Urinary tract infections	Because SGLT2 inhibitors prevent excess glucose from being reabsorbed in the kidneys, glucose is excreted in the urine. This glycosuria creates a favorable environment for bacteria and fungi in the genitourinary tract, increasing the risk of infections in patients using these medications. This should be closely monitored in subsequent follow-ups [55].
Increased risk of amputations (specifically canagliflozin)	Preliminary data from the CANVAS program has raised concerns about a potentially increased risk of lower limb amputations concurrent with the use of SGLT2 inhibitors, especially canagliflozin. This remains a serious consideration in patients with peripheral artery diseases, prior amputations, or other high-risk features. Therefore, careful patient selection and monitoring are advised when prescribing these medications [56].

## Data Availability

Not applicable.
